# Structural and functional insights into the C-terminal signal domain of the Bacteroidetes type-IX secretion system

**DOI:** 10.1098/rsob.230448

**Published:** 2024-06-12

**Authors:** Danuta Mizgalska, Arturo Rodríguez-Banqueri, Florian Veillard, Mirosław Książęk, Theodoros Goulas, Tibisay Guevara, Ulrich Eckhard, Jan Potempa, F. Xavier Gomis-Rüth

**Affiliations:** ^1^ Department of Microbiology, Faculty of Biochemistry, Biophysics and Biotechnology, Jagiellonian University, Kraków, Poland; ^2^ Proteolysis Laboratory, Department of Structural Biology, Molecular Biology Institute of Barcelona (CSIC), Barcelona, Catalonia 08028, Spain; ^3^ Department of Food Science and Nutrition, School of Agricultural Sciences, University of Thessaly, Karditsa 43100, Greece; ^4^ Synthetic Structural Biology Group, Department of Structural Biology, Molecular Biology Institute of Barcelona (CSIC), Barcelona, Catalonia 08028, Spain; ^5^ Department of Oral Immunology and Infectious Diseases, University of Louisville School of Dentistry, Louisville, KY 40202, USA

**Keywords:** periodontal disease, bacterial virulence factor, infectious disease, protein secretion, X-ray crystal structure, T9SS

## Abstract

Gram-negative bacteria from the Bacteroidota phylum possess a type-IX secretion system (T9SS) for protein secretion, which requires cargoes to have a C-terminal domain (CTD). Structurally analysed CTDs are from *Porphyromonas gingivalis* proteins RgpB, HBP35, PorU and PorZ, which share a compact immunoglobulin-like antiparallel 3+4 β-sandwich (β1–β7). This architecture is essential as a *P. gingivalis* strain with a single-point mutant of RgpB disrupting the interaction of the CTD with its preceding domain prevented secretion of the protein. Next, we identified the C-terminus (‘motif C-t.’) and the loop connecting strands β3 and β4 (‘motif Lβ3β4’) as conserved. We generated two strains with insertion and replacement mutants of PorU, as well as three strains with ablation and point mutants of RgpB, which revealed both motifs to be relevant for T9SS function. Furthermore, we determined the crystal structure of the CTD of mirolase, a cargo of the *Tannerella forsythia* T9SS*,* which shares the same general topology as in *Porphyromonas* CTDs. However, motif Lβ3β4 was not conserved. Consistently, *P. gingivalis* could not properly secrete a chimaeric protein with the CTD of peptidylarginine deiminase replaced with this foreign CTD. Thus, the incompatibility of the CTDs between these species prevents potential interference between their T9SSs.

## 1. Introduction

The interface between bacteria and their extracellular environment is permeated by secretion machineries, which are multiprotein gateways for the transport of cargoes from the cytosol across the cytoplasmic inner membrane (IM; ‘protein export’) and, when present, outer membrane (OM; ‘protein secretion’) [1–4]. These systems are critical for bacterial viability at a site of infection or colonization as they serve nutrient acquisition and communication with other bacteria, often across species barriers [2,3]. They further enable bacterial adhesion to biofilms, S-layer formation and gliding motility, as well as the secretion of virulence factors to disarm host defences and competing bacteria at the site of colonization/infection [3–5]. One of the 11 secretion systems [3] that are currently known to exist is the type-IX secretion system (T9SS) [6], which was earlier known as the ‘PerioGate’ and ‘Por Secretion System’ [7–9]. It is widely found in the Gram-negative Bacteroidetes (synonymous with Bacteroidota) phylum of the ‘FCB superphylum’ [10], and sequences of T9SS core proteins have been identified in the genomes of 90 species of this phylum, with the notable exception of *Bacteroides* spp. [11]. T9SSs may also be present in other FCB phyla [12], including Chlorobiota, Ignavibacteriota, Rhodothermota, Gemmatimonadetes, Cloacimonadota and Fibrobacterota [6,11,13–15]. Remarkably, T9SS genes are absent from other bacterial phyla, archaea and eukaryotes [11].

T9SSs or their component proteins have been experimentally studied from *Candidatus paraporphyromonas polyenzymogenes; Capnocytophaga ochracea; Cellulophaga algicola* and *Cellulophaga omnivescoria; Cytophaga hutchinsonii; Dyadobacter* sp.; *Fibrobacter succinogenes; Flavobacterium columnare*, *Flavobacterium johnsoniae*, *Flavobacterium collinsii*, *Flavobacterium spartansii* and *Flavobacterium psychrophilum; Parabacteroides distasonis; Prevotella intermedia* and *Prevotella melaninogenica; Porphyromonas gingivalis; Riemerella anatipestifer; Roseithermus sacchariphilus; Sporocytophaga myxococcoides; Tannerella forsythia;* and *Tenacibaculum maritimum* [4,7,9,11,14,16–35]. Many of these species infect animals, among which *P. gingivalis* and *T. forsythia* are human periodontopathogens that proliferate in the gingiva under dysbiotic conditions of the oral microbiome. Together with *Treponema denticola*, they form the ‘red complex’ [36] that causes grievous gingivitis and periodontal disease, which affects an estimated 750 million people worldwide in its severe form and is the sixth most prevalent disabling health condition [37–40].

The T9SS is a specialized OM shuttle of protein cargoes that includes a rotary motor, a translocon, an OM-associated scaffold and an attachment complex [4,41]. It operates in conjunction with the general secretory (*Sec*) pathway for the previous export of cargoes from the cytosol across the IM [4,41]. Once in the periplasm, the N-terminal *Sec* signal peptide is removed, and cargoes fold and are escorted to the T9SS translocon for secretion. The best-characterized T9SSs are from *F. johnsoniae* and *P. gingivalis* [4,7,9,28]. In the latter, it is the major protein secretion pathway and is conformed or regulated by at least 24 ‘Por’ proteins [4,9,41]. It secretes >35 cargo proteins, which include essential virulence factors for infection such as the gingipain cysteine peptidases Kgp, RgpA and RgpB [42]; *Porphyromonas* peptidylarginine deiminase (PPAD) [8,43]; a 70 kDa metallocarboxypeptidase (CPG70) [44]; and a 35 kDa haemin-binding protein (HBP35) [27].

T9SS transport requires a C-terminal domain (CTD) of 70–100 residues, also known as the ‘T9SS signal’ [16,17,41,45–50], which contrasts with the short unstructured signal peptides of other secretion systems [2,51]. In fact, all Bacteroidetes species potentially encompassing a T9SS also encode putative cargo proteins featuring a CTD and, vice versa, species lacking T9SS genes also miss-predicted proteins with a CTD [11]. Thus, T9SS cargoes are also dubbed ‘CTD proteins’ [46,52]. In *P. gingivalis*, the CTD is generally chopped off during or after OM translocation by the C-terminal T9SS signal peptidase PorU [53], which operates within an ‘attachment complex’ with PorQ, PorV and PorZ [4,41,54], and cargoes are released to the environment. In a subset of them, however, anionic lipopolysaccharide provided by PorZ is attached to the new C-terminus by PorU, which thus also acts as a sortase-type transpeptidase [53,55,56]. These cargoes are anchored to the extracellular side of the OM [17,57–60]. Notably, some T9SS constituents that are themselves secreted, such as PorA, PorU and PorZ, also possess a CTD, which, however, is not removed upon secretion [5,41,61]. Thus, there are PorU-processed and PorU-unprocessed CTDs.

The CTD is apparently necessary and sufficient for T9SS secretion [4,11,52,62]. Moreover, CTDs are functionally exchangeable between cargoes within a given species, as shown for *P. gingivalis* Kgp with the CTD of RgpB [45]. What is more, the CTDs of *P. gingivalis* cargo proteins RgpB, PPAD, CPG70 or P27 fused to a green fluorescent protein caused the resulting chimaeras to be secreted and anchored to the OM by this bacterium [45,52]. In *F. johnsoniae*, the same fluorescent protein and mCherry, furnished with the CTD of the RemA adhesin [11] or the ChiA chitinase [47], were secreted by the T9SS. CTDs may also function across species within Bacteroidetes, as shown for the *Flavobacteriia* class. Indeed, *C. algicola* AmyA α-amylase was secreted by the T9SS of *F. johnsoniae* [11]. Across classes, the latter translocon recognized the CTD of *C. hutchinsonii* (class Cytophagia) Cel9B cellulase but not that of *P. gingivalis* (class Bacteroidia) RgpB [11]. Moreover, the CTD of *C. hutchinsonii* Cel9A cellulase is required to be *N*-glycosylated in the periplasm prior to secretion and cell-surface exposure in what resembles an additional step of regulation in this species [63]. Overall, these findings pinpoint species-specific differences across the T9SS signals and disparate interspecific compatibility.

Although CTDs have been classified into types A, B and C, sequence identities are generally very low and there are no obvious motifs attributable to cargo translocation and/or cleavage through the C-terminal T9SS signal peptidase [4,11]. This has fuelled the hypothesis that conserved structural elements rather than sequence stretches may be responsible for function [11]. In this sense, the structures of the CTDs of RgpB [49,64] and HBP35 [27], which are removed upon secretion, as well as those of the non-cleaved CTDs of PorZ [5] and PorU [41], have been determined. All correspond to *P. gingivalis* proteins and no CTDs from other species have been reported to date.

Here, we sought to identify molecular determinants for T9SS secretion and PorU-cleavage in *P. gingivalis* by comparing the four aforementioned structures with two ad hoc high-confidence homology models and verified them with a cohort of mutant strains that were subjected to cellular and functional assays. Furthermore, we determined the crystal structure of the CTD of the *T. forsythia* T9SS cargo mirolase [65] (dubbed ‘LKK’; from L^706^ to K–K^791^, see UniProt entry (UP) A0A0A7KVG3 for residue numbering), the first outside *P. gingivalis*, and assessed if the above structural determinants were conserved across species. Finally, we checked the CTD compatibility of *T. forsythia* and *P. gingivalis*.

## 2. Results and discussion

### 2.1. Molecular determinants of *P. gingivalis* C-terminal domains for T9SS function

**Figure 1.**
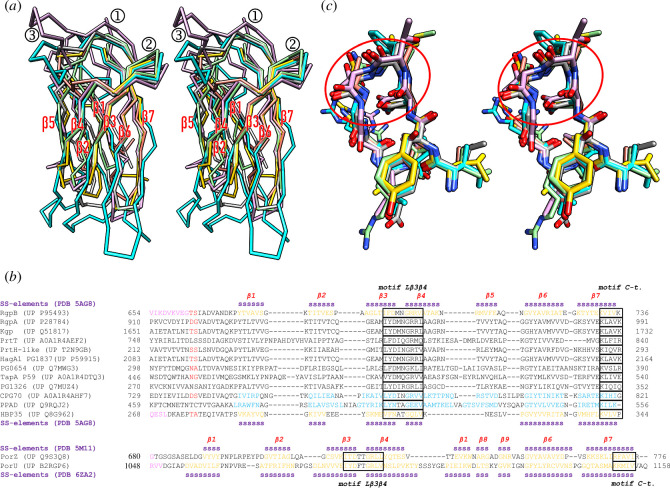
Structural comparison of *P. gingivalis* CTDs. (*a*) Overall superposition in cross-eye stereo of the Cα-traces of the non-cleaved CTDs of PorU (Protein Data Bank (PDB) access code 6ZA2; A^1054^–Q^1158^, for UP codes, see (*b*); cyan) and PorZ (PDB 5M11; E^688^–R^776^; plum), as well as of the cleaved CTDs of RgpB (PDB 5AG8; K^672^–K^736^; light green), HBP35 (PDB 5Y1A; P^287^–P^344^; salmon), CPG70 (predicted model; Q^746^–G^821^; gold) and PPAD (predicted model; K^476^–K^556^; dark grey). The seven consensus strands are labelled β1–β7, as well as the two essential structural motifs for T9SS function (①, motif C-t. and ②, motif Lβ3β4) and the non-functional β-hairpin within Lβ5β6 (③). (*b*) Structure-based sequence alignments of the cleaved CTDs of selected cargoes (top alignment) and the non-cleaved CTDs of PorZ and PorU (bottom alignment). The respective UP access codes and flanking residue numbers are provided. Residues in β-strand conformation (‘SS-elements’) according to the respective PDB entries for RgpB and PorZ (above the respective alignment block) and HBP35 and PorU (below the respective alignment block) are displayed in orange. They are earmarked with a magenta ‘s’ and labelled β1–β7. PorU and PorZ have an extra β-ribbon (strands β8+β9) inserted after the fifth strand. Predicted strand residues of the two homology models (CPG70 and PPAD) are in blue. The last residues of the respective upstream domains of structurally analysed proteins are shown in magenta. Determined or putative cleavage sites by PorU are flanked by residues in red [50,53,55]. Motifs Lβ3β4 and C-t. are boxed and are similar to regions B and E of [46]. (*c*) Superposition in stereo of motif Lβ3β4 (red ellipse, end-on view) from PorU (residues I^1090^–V^1098^, carbons in cyan), PorZ (I^720^–L^728^, plum), RgpB (I^694^–V^702^, light green), HBP35 (V^287^–V^295^, tan), CPG70 (L^773^–V^781^, gold) and PPAD (L^505^–V^513^, grey). The residue numbers correspond to the respective UP entries in (*b*). The orientation results from that of (*a*) after successive vertical and horizontal rotations of 120°C and 40°C, respectively.

We compared the CTD structures of PorZ [5], PorU [41], HBP35 [27] and RgpB [49] with the calculated high-confidence comparative models (see §3.10) of the CPG70 and PPAD CTDs (figure 1*a*). We found that the general topology and connectivity, as first described for RgpB [49], were maintained. All molecules consisted of a central immunoglobulin-like moiety [66–68], which features an antiparallel seven-stranded β-sandwich with a four-stranded (β4–β3–β6–β7) and a three-stranded (β5–β2–β1) β-sheet, with intersheet angles of ~30–40°C and Greek-key strand connections (figure 1*a*). However, with the exception of the loop connecting strands β3 and β4 (Lβ3β4), each structure displayed large variations in the length of the strands and the connecting loops, which led to rather high *rmsd* and low sequence-identity values in pairwise comparisons (table 1). We then performed a structure-assisted sequence alignment and included a total of 12 CTDs from reported *P. gingivalis* cargoes, which confirmed the lack of significant sequence similarity (figure 1*b*). We found that PorU-processed cargoes are generally shorter and span 67–88 residues from the predicted/determined PorU cleavage site [50,53,55] to the C-terminus, while PorZ and PorU encompass 96 and 108 residues, respectively. Thus, CTDs significantly vary in length.

**Table 1.** Pairwise comparison of experimental and predicted *P. gingivalis* CTD structures.

	PorU	PorZ	RgpB	HBP35	PPAD
PorZ	80; 105/89; 3.0; 6				
RgpB	64; 105/65; 3.0; 6	61; 89/65; 1.8; 20			
HBP35	29; 105/59; 2.8; 0	54; 89/59; 2.3; 22	57; 65/59; 2.0; 30		
PPAD	77; 105/81; 1.9; 13	72; 89/81; 2.0; 10	59; 65/81; 2.7; 8	54; 59/81; 2.6; 7	
CPG70	72; 105/76; 2.4; 15	70; 89/76; 2.2; 13	60; 65/76; 2.1; 27	56; 59/76; 2.7; 9	72; 81/76; 1.9; 18

For each pair of structures, the number of aligned residues out of the total residues of each protein, the overall *rmsd* (in Å) and the sequence identity (in %) are indicated.

**Figure 2.**
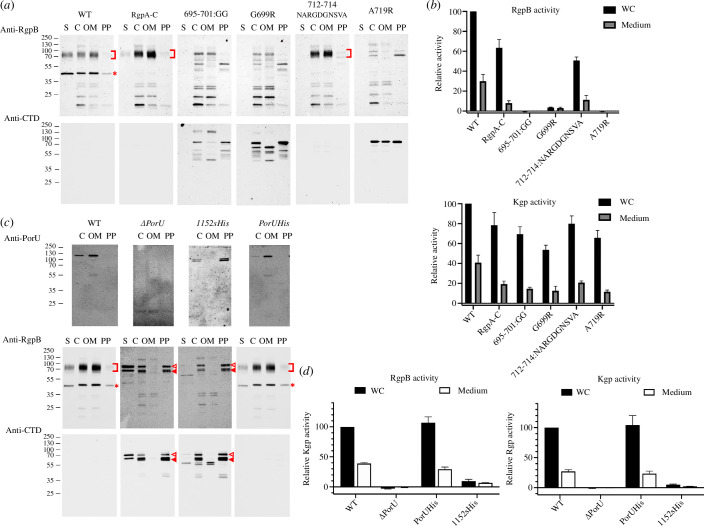
Cell-fraction analysis and proteolytic activity of RgpB and PorU mutant strains. (*a*) Western blotting analysis of RgpB-mutant strains in the supernatant (S), whole-cell extract (C), outer-membrane (OM) fraction and periplasmic/cytoplasmic fraction (PP) employing polyclonal antibodies against RgpA and RgpB (top row, pAb GP1) or the CTD of RgpB (bottom row). Red square brackets pinpoint membrane-type RgpB and asterisks denote the isolated RgpA catalytic domain. The latter is missing in the RgpA-C strain and the mutants generated with a RgpA-C template. The absence of gingipain activity results from deficient proteolytic processing of the respective zymogens, which require the entire prodomain to be removed and degraded to release activity [56]. (*b*) RgpB (top panel) and Kgp (bottom panel) activity relative to the wild-type (100%) of the RgpB-mutant strains of (*a*) in whole-cell (WC) cultures and the culture medium after cell centrifugation. Experiments were performed in triplicate. (*c*) Western blotting analysis of PorU-mutant strains employing the monoclonal antibody 7G9 against PorU (top row) and the polyclonal antibodies of (*a*) against RgpB and its CTD (middle and bottom rows). Red square brackets pinpoint membrane-type RgpB; asterisks denote the isolated RgpA catalytic domain; open triangles hallmark the unprocessed, full-length 80 kDa RgpB zymogen including the intact N-terminal prodomain and the CTD; and full triangles designate the N-terminally truncated RgpB zymogen. (*d*) RgpB (left panel) and Kgp (right panel) activity of the PorU-mutant strains of (*c*) in whole cultures (WC) and the culture medium after cell centrifugation.

Previous studies with a cohort of 16 point mutations across the CTD of RgpB had revealed no particular residue side chain indispensable for export [69,70]. Thus, we hypothesized that an intact well-folded CTD and, possibly, particular three-dimensional structural motifs would be required for secretion and PorU-mediated cleavage/transpeptidation, as previously suggested [11]. To validate the importance of overall CTD integrity, we identified RgpB residue A^719^ (numbering according to UP P95493), which is the central residue of the penultimate β-strand β6 of the CTD and is engaged in interactions with the upstream protein domain [49]. We generated a *P. gingivalis* strain with the alanine replaced with arginine (mutant A^719^R). To perform such RgpB mutations, we routinely employ the RgpA-deficient mutant strain RgpA-C to prevent the highly homologous *rgpA* gene from interfering with the genetic manipulation of *rgpB* and RgpB protein detection [16,71]. We compared the occurrence of the mutant protein in distinct cell-culture fractions and extracellular gingipain activity with those of the wild-type and RgpA-C as positive controls. We found that the mutant protein was not secreted to the OM but accumulated in the periplasm (figure 2*a*), and the strain lacked extracellular RgpB proteolytic activity (figure 2*b*). This is indicative of T9SS malfunction for this cargo [16,41,56]. In contrast, Kgp activity was unaffected, which documents the correct function of the T9SS for other cargoes (figure 2*b*).

As to particular CTD elements that may be relevant for T9SS function, we identified two structural motifs, ‘motif C-t.’ and ‘motif Lβ3β4’ (figure 1*a,b*). They are conserved and located on the surface, near the top edge of the domain (figure 1*a*,*c*) and, thus, potentially accessible for interaction with the T9SS translocon and subsequent PorU cleavage [41].

### 2.2. Motif C-t.

Previous studies have underlined the importance of the CTD C-terminal end for function [11]. In particular, the truncation of two residues—but not one—at the C-terminus of RgpB created an inactive variant that is retained in the periplasm [16]. Moreover, the T9SS targeting signal of HBP35 was localized to the last 22 residues of the CTD [52]. Inspection of the superposed CTD structures revealed that the main chains of the C-terminal β-strand coincide (figure 1*a*), which suggests they may be important for structural integrity and/or function of the whole domain. Moreover, a conserved lysine is found in equivalent positions of strand β7 (figure 1*b*).

To validate this hypothesis, we constructed and assayed *P. gingivalis* strains featuring two PorU mutants, in which the last six residues were replaced with hexahistidine (replacement mutant 1152sHis) or eight histidines were added to the C-terminus (extension mutant PorUHis). By using the wild-type and a PorU deletion mutant (ΔPorU) as positive and negative controls, respectively, we found that in the PorUHis mutant, PorU and RgpB were correctly translocated to the OM surface and the CTD of RgpB was properly removed by PorU, as found in the wild-type (figure 2*c*). Moreover, the mutant evinced extracellular RgpB and Kgp activity indistinguishable from the wild type (figure 2*d*). In contrast, mutant 1152sHis mimicked the ΔPorU deletion mutant in that RgpB was not exported or processed correctly, so its CTD was still attached (figure 2*c*). Moreover, mutant PorU protein accumulated in the periplasm. Finally, again, as in ΔPorU, no relevant extracellular RgpB or Kgp activity was detected (figure 2*d*). Overall, these results indicate general malfunctioning of the T9SS due to aberrant PorU.

Altogether, the findings of the two mutants confirm the importance of the residues constituting the C-terminal motif for translocation, but the C-terminus may be extended without consequences. This is consistent with reports for PorZ, for which elongation of the C-terminus by histidine tags likewise did not cause functional T9SS impairment, but replacement of the last residues with histidines did [5]. Furthermore, extension of the C-terminus by polyhistidine tags, as well as deletion of the last two residues—but not four residues—did not affect the secretion and OM attachment of PPAD. In fact, the CTDs of PorZ and PorU evince one and three extra C-terminal residues, respectively, when superposed onto those of the other cargoes (figure 1*a,b*).

### 2.3. Motif Lβ3β4

All CTD models showed an excellent fit of the segment encompassing Lβ3β4 (figure 1*a,c*), which suggests a possible consensus role in T9SS function. This finding is consistent with a previous deletion mutant of RgpB lacking L^692^–V^702^, which suppressed the secretion of this cargo [69]. However, as this mutant had been designed before the structure of the RgpB CTD was available [49], we hypothesized that an internal deletion of 11 residues ablating not only the connecting loop but also most of strands β3 and β4 might have caused the collapse of the entire domain instead of selectively ablating a specific structural element in an otherwise intact scaffold. Thus, we obtained a more conservative structure-based deletion strain that should keep the overall structure well folded, in which F^695^–D–M–N–G–R–R^701^ was replaced with two glycines (mutant 695–701:GG). We found that in this mutant, RgpB accumulated in the periplasm and was not secreted and that extracellular RgpB activity—but not Kgp activity—was significantly diminished (figure 2*a,b*). Moreover, the aforementioned study by Slakeski *et al*. also reported that replacement in RgpB of the only conserved residue of the Lβ3β4 motif, a glycine (G^699^) in the first position of the fourth strand (figure 1*b*), with proline had no significant deleterious effect [69]. We hypothesized that this replacement may not have had a structural impact large enough to cause motif disruption. To verify this, we replaced this glycine with arginine (mutant G^699^R) and found that this mutation actually hindered RgpB secretion and processing to its active mature form (figure 2*a*). Moreover, extracellular RgpB activity was ablated (figure 2*b*). Thus, this glycine is functionally relevant and pinpoints motif Lβ3β4, narrowed down to the encircled region in figure 1*c*, as essential for CTD recognition and cargo secretion by the T9SS translocon.

### 2.4. The β-hairpin within Lβ5β6 of PorU and PorZ is functionally not relevant

Upon examining PorU-processed and PorU-unprocessed CTDs for any noticeable variations, we found that PorU and PorZ share an additional β-hairpin at the end of the fifth strand, which is not present in the other four models (figure 1*a*,*b*). This protrusion, which accounts for most of the extra residues in the two Por proteins when compared with PorU-processed CTDs, should hamper any functional interaction Lβ5β6 might perform, so we hypothesized that this element might be engaged in PorU binding for cargo processing within the attachment complex. To validate this hypothesis, we constructed a mutant strain in which E^712^–A–Q^714^ of RgpB was replaced with N–A–R–G–G–D–G–N–S–V–A (mutant 712–714:NARGGDGNSVA). This mimics the insertion of PorZ except for a serine replacing an arginine (figure 1*b*). Unexpectedly, we found that in this mutant RgpB was correctly exported, cleaved by PorU and anchored on the surface in a manner indistinguishable from the RgpA-C positive control (figure 2*a*), and extracellular gingipain activity was not affected (figure 2*b*). Thus, motif Lβ5β6 is apparently not relevant to distinguish between translocation and PorU processing, so the discerning features between these two functions remain to be determined.

### 2.5. Crystal structure of a C-terminal domain orthologue from *T. forsythia*

**Figure 3.**
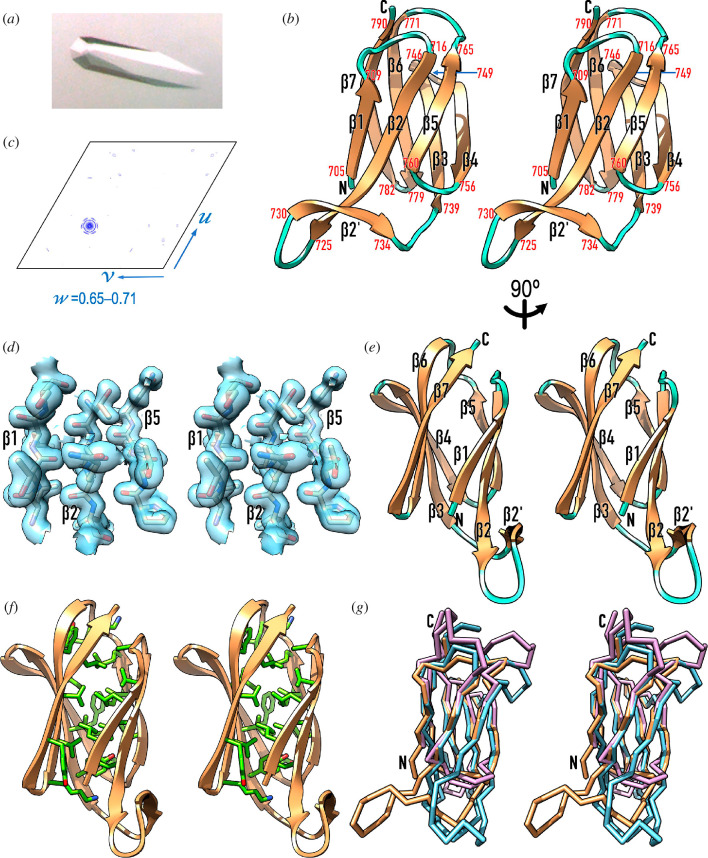
Crystallographic studies of *T. forsythia* LKK. (*a*) Trigonal crystal of space group P3_1_ of selenomethionine-derivatized LKK diffracting to 1.6 Å resolution. (*b*) Native Patterson map section (axes *u*, *v*, *w*) viewed down the *w* axis for fraction 0.65–0.71. A peak of 72% the height of the origin peak at fractional coordinates 0.331, 0.663 and 0.678 accounts for strong translational non-crystallographic symmetry, which obscures hemihedral twinning following twin law h,–h–k,–l (estimated twin fraction *α* = 0.423). (*c*) Ribbon-type plot in cross-eye stereo of LKK, which encompasses eight β-strands as salmon arrows (β1–β7 *plus* β2’) connected by coil regions in cyan. The flanking residues of each strand are numbered in red, and the N- and the C-terminus are labelled in black. In this view, the molecule has been rotated vertically ~180° with respect to figure 1*a* for clarity. (*d*) Detail of the final σ_A_-weigthed (2*mF*_obs_–*DF*_calc_)-type Fourier map, contoured at 1*σ* above the threshold, centred on the three β-strands (β1, β2 and β5) of the front sheet of molecule A. The final refined model is shown for segments L^706^–S^709^, T^718^–L^721^ and P^759^–Q^762^, approximately in the view of (*c*). (*e*) Same as (*c*) after a vertical 90° rotation. (*f*) Variant of (*e*) depicting the side chains that form the hydrophobic core of the LKK moiety, with carbons in light green. The participating residues are L^706^, L^708^, A^713^, V^717^, L^719^, L^721^, Y^740^, I^742^, I^744^, F^754^, T^756^, F^761^, I^763^, P^764^, M^765^, L^768^, Y^773^, V^775^, V^777^, K^779^, Y^784^, L^788^ and K^790^. (*g*) Superposition of LKK as Cα-plot in sandy brown in the view of (*c*) onto the structures of the PorU (cyan; PDB 6ZA2) and PorZ (plum; PDB 5M11) CTDs.

The LKK protein domain from *T. forsythia* mirolase was recombinantly produced in *Escherichia coli* with selenomethionine replacing methionine as an N-terminal fusion with glutathione-S-transferase, and purified by affinity and size-exclusion chromatography steps (see §3.7). After tag removal, the protein was crystallized in the form of well-diffracting trigonal crystals (see figure 3*a* and table 2). However, structure solution and refinement proved exceptionally difficult owing to the simultaneous occurrence of translational non-crystallographic symmetry (tNCS; figure 3*b*) and hemihedral twinning, which led to space-group ambiguity (see §3.9). Eventually, the structure was solved in the primitive space group P3_1_21 by single-wavelength anomalous diffraction (SAD) with data collected at the selenium K-edge, but it could only be refined in P3_1_ after the diffraction data had been detwinned (table 2). All this notwithstanding, the final structure (figure 3*c*), with six protomers in the crystallographic asymmetric unit (a.u.), was backed by solid Fourier map density (figure 3*d*) and exhibited good validation parameters (table 2).

**Table 2.** Crystallographic data.

dataset	LKK (*T. forsythia* mirolase CTD, SeMet)
beam line (synchrotron)	ID23-1 (ESRF)
detector	ADSC Quantum Q315r
space group / protomers per a.u.^a^	P3_1_ / 6 (A–F)
cell constants (*a* and *c*, in Å)	81.45, 66.55
wavelength (Å)	0.91917
no. of measurements / unique reflections	731 662 / 65 076
resolution range (Å) (outermost shell)^b^	48.4–1.60 (1.70–1.60)
completeness (%)	99.8 (99.6)
R_merge_^c^	0.121 (0.954)
R_meas_^c^	0.127 (0.999)
CC(^1^/_2_)^c^	0.999 (0.943)
Average intensity^d^	9.0 (2.7)
B-factor (Wilson) (Å^2^)	23.5
aver. multiplicity	11.2 (11.1)
twinning law	h, -h-k, -l
twinning fraction *α*	0.423
no. of reflections used in refinement [in test set]^e^	64 231 [656]
crystallographic R_factor_ / free R_factor_	0.275 / 0.302
F_obs_ , F_calc_ correlation [test set]	0.933 [0.930]
no. of protein residues / atoms / solvent molecules / non-covalent ligands	518 / 4048 / 476 / 2 K^+^, 6 Cl^−^, 6 PEG
Rmsd from target values	
bonds (Å) / angles (°)	0.008 / 0.97
average B-factors (Å^2^): overall // mol. A/B/C/D/E/F	21.7 // 17.9/22.9/22.2/17.8/22.8/22.0
all-atom contacts and geometry analysis^f^	
protein residues	
in favoured Ramachandran regions / outliers / all residues	503 (98.4%) / 0 / 511
with outlying rotamers / bonds / angles / chirality / torsion	5 (1.1%) / 0 / 0 / 0 / 0
all-atom clashes / clashscore	20 / 2.2
PDB access code	8QB1

^a^ Abbreviations: a.u., crystallographic asymmetric unit; PEG, diethylene glycol.

^b^ Values in parentheses refer to the outermost resolution shell.

^c^ For definitions, see [72].

^d^ Average intensity is < I/σ(I) > of unique reflections after merging according to *Xscale* [73].

^e^ Refinement was performed against processed data that were subsequently detwinned according to twin-law (h,-h-k,-l) and a twinning factor *α* = 0.423 with program *Detwin* within *Ccp4*.

^f^ According to the wwPDB Validation Service: https://wwpdb-validation.wwpdb.org/validservice.

The LKK moiety has an elongated ellipsoidal shape of ~42 Å height, ~26 Å width and ~22 Å depth in the view of figure 3*c*. As the aforementioned *P. gingivalis* CTDs, it is an antiparallel β-sandwich with a three-stranded sheet (β1, β2 and β5, from left to right in figure 3*c*) and a four-stranded sheet (β7, β6, β3 and β4). Two extra N-terminal residues from the purification tag (glycine and serine) replace domain residues L^704^ and Y^705^ and form the first residues of strand β1, so that the domain actually spans 88 residues (L^704^–K^791^). This lies within the range of cleavable *P. gingivalis* CTDs (see §2.1). The two LKK sheets are arranged with an intersheet angle of ~35° and have Greek-key connectivity (figure 3*c,e*). The strands are connected by short loops spanning either two residues (Lβ3β4 and Lβ6β7), three residues (Lβ4β5), five residues (Lβ5β6) or six residues (Lβ1β2). The only exception is Lβ2β3. Here, a further strand external to the central sandwich (β2′) interacts with the elongated tip of strand β2, which gives rise to a 13-residue connector that generates a hairpin-like appendage at the bottom of the sandwich (figure 3*c,e*). Overall, the moiety is cohered by an internal hydrophobic core made up by 23 residues (figure 3*f*). Finally, the C-terminal segment of strand β7 encompasses the sequence K^787^–L–I–K–K^791^, which is found at the C-terminus of five unique serine- and metal-dependent peptidases secreted by the T9SS of *T. forsythia* in addition to mirolase, collectively named ‘KLIKK-peptidases’ [65,74]. This conservation is in line with the above sequence requirements of motif C-t. in *P. gingivalis,* which also includes a conserved lysine in the middle of C-terminal strand β7 (see §2.2).

Structure similarity searches with LKK identified the CTDs of PorZ (Z-score 10.0, *rmsd* 1.7 Å, 72 aligned residues, 18% identity; see [75]) and PorU (Z-score 9.9, *rmsd* 1.9 Å, 78 aligned residues, 19% identity) as the closest structural relatives. Contrary to the *Tannerella* moiety, these *Porphyromonas* CTDs correspond to non-cleaved forms. Notably, the similarity was significantly lower with the cleavable CTDs of RgpB (Z-score 8.3, *rmsd* 2.2 Å, 72 aligned residues, 7% identity) and HBP35 (Z-score 6.0, *rmsd* 2.2 Å, 54 aligned residues, 17% identity). Superposition of LKK onto the PorU and PorZ CTDs (figure 3*g*) revealed that the topology and connectivity of the sandwich β-strands are maintained but loops largely deviate and pairwise sequence identities span mere 18–19%. In particular, the extended loop Lβ2β3 of LKK is much longer and adopts completely different trajectories in the Por proteins. In contrast, Lβ6β7 and Lβ5β6 are shorter in LKK. The latter is uniquely longer in both PorU and PorZ but is apparently not relevant for function (see §2.4).

### 2.6. C-terminal domain interchange between *P. gingivalis* and *T. forsythia*

**Figure 4.**
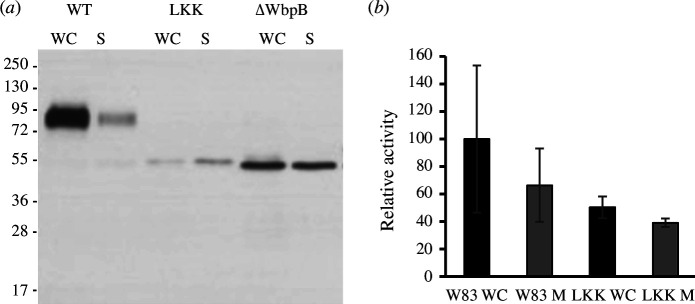
Cross-species CTD exchange between *P. gingivalis* and *T. forsythia.* (*a*) Western blotting analysis of PPAD export in WC cultures and the culture medium (S) for the wild-type protein (labelled WT) and a PPAD chimaera with LKK as CTD (labelled LKK), as well as for a *P. gingivalis* mutant strain (ΔWbpB), which does not attach lipopolysaccharide and thus does not anchor substrates to the OM. The chimaera mimics the phenotype of the latter strain, though with a substantially lower yield, which indicates that the protein is not properly secreted. (*b*) PPAD activity in whole-cell extract and medium for the wild-type protein and the chimaera.

We found that, in addition to the much longer Lβ2β3, LKK lacked one residue in motif Lβ3β4, which is relevant for T9SS function in *P. gingivalis* (see §2.3), and the conserved glycine was replaced with methionine. Thus, we anticipated specific differences between the T9SSs of the two species, as previously reported for the *P. gingivalis* and *F. johnsoniae* pair [11]. We constructed a strain with a replacement mutant of *P. gingivalis* PPAD, in which the CTD (A^474^–K^556^; numbering according to UP Q9RQJ2) was replaced with LKK (D^702^–K^791^) to yield mutant PPAD-LKK. We found that this chimaera was not properly secreted and mimicked a strain that cannot anchor cargoes to the OM (figure 4*a*). Moreover, the mutant strain evinced substantially less extracellular PPAD activity (figure 4*b*). This confirms that the CTDs are not functionally interchangeable between *P. gingivalis* and *T. forsythia* due to the disruption of motif Lβ3β4, despite both species belonging to the Bacteroidia class.

### 2.7. Conclusion

In an effort to determine molecular determinants for the recognition of the specific T9SS secretion signal, a folded CTD, by the translocon in *P. gingivalis*, as well as for its cleavage by the C-terminal signal peptidase, we compared six structures. We found the domain’s C-terminus and loop Lβ3β4 to be essential for function. In contrast, we could not determine the distinguishing features between Por-cleaved and PorU-non-cleaved CTDs.

Given that all the reported CTD structures are from *P. gingivalis*, we determined the structure of a CTD from a *T. forsythia* cargo, LKK. This module shares the gross architecture with its *P. gingivalis* counterparts but lacks the specific features of Lβ3β4, and it evinces a substantially longer Lβ2β3. Consistently, the replacement of the CTD of a *P. gingivalis* cargo with LKK hampered its secretion, which points to specific differences in CTD recognition between the translocons of these two species. This may prevent potential functional interference due to the accidental transfer of cargo genes between two phylogenetically close periodontopathogens, which share the human gingival crevice as an ecological niche, are found in the same biofilm, and compete for resources and thrive at the site of infection [76].

## 3. Material and methods

### 3.1. Bacterial culturing

All *P. gingivalis* mutants were derived from strain W83 (see table 3 for a list of strains and cells used in this study) and were cultured in trypticase soy broth (30 g l^−1^) enriched with haemin (5 mg l^−1^), menadione (2 mg ml^−1^), ʟ-cysteine (0.5 g l^−1^) and yeast extract (5 g l^−1^). Solid cultures were additionally supplemented with 5% defibrinated sheep blood, 1.5% agar, and, when antibiotic selection was needed, erythromycin (5 μg ml^−1^ or tetracycline (1 μg ml^−1^). Cells were cultivated in an incubator from Don Whitley Scientific at 37°C under anaerobic conditions (10% carbon dioxide, 5% hydrogen and 85% nitrogen). For molecular cloning procedures, *E. coli* strain DH5α was grown in Luria–Bertani medium (Lennox). Solid cultures in the same medium were prepared with 1.5% agar and supplemented with ampicillin (100 μg ml^−1^) for antibiotic selection.

**Table 3.** List of strains and plasmids.

strains	description (***genotype;*** resistance)	source/reference
*E. coli* DH5α	general cloning host	Thermo Fisher
*E. coli* BL21 (DE3)	expression of PorU protein	Millipore
*P. gingivalis* W83		
*WT*	wild type	
∆PorU	*porU::ermF;* Em^r^	[5]
1152sHis	*porU1153-1158::6His, ermF;* Em^r^	this study
PorUHis (1158iHis)	*porU1158ins8His, ermF;* Em^r^	[41]
RgpA-C	*RgpA::cat;* Cm^r^	[16]
695-701:GG	*RgpA::cat, RgpB 695-701::GG, tetR,* Cm^r^ ,Tet^r^	this study
G699R	*RgpA::cat, RgpB G699R, tetR;* Cm^r^, Tet^r^	this study
A712-714:NARGDGNSVA	*RgpA*::cat*, RgpB 712-714::NARGDGNSVA, tetR;* Cm^r^,Tet^r^	this study
A719R	*RgpA::cat, RgpB A719R, tetR;* Cm^r^, Tet^r^	this study
PPAD-LKK	*PPAD 474-556::Mirolase 702:791, tetR;* Tet^r^	this study
∆WbpB	*wbpB::tetQ*; Tet^r^	this study
plasmids		
p22-E-1158iHis	suicide plasmid for PorUHis (1158iHis) strain generation	[41]
pPorU 1152sHis	suicide plasmid for 1152sHis strain generation	this study
pRgpB-t	suicide plasmid for RgpB-coding gene modification	[16]
pRgpB 695-701:GG	suicide plasmid for 695-701:GG strain generation	this study
pRgpB G699R	suicide plasmid for G^699^R strain generation	this study
pRgpB 712-714:NARGDGNSVA	suicide plasmid for 712-714:NARGDGNSVA strain generation	this study
pRgpB A719R	suicide plasmid for A719R strain generation	this study
pPPAD-tet	suicide plasmid for PPAD-coding gene modification	this study
pPPAD-LKK	suicide plasmid for PPAD-LKK strain generation	this study
LKK-pGEX6-P-1	expression plasmid for LKK domain	this study
pWbpB-t	suicide plasmid for *wbpB* gene deletion	this study

### 3.2. Subcellular fractionation of *P. gingivalis* cells

*P. gingivalis* cultures (controls and mutants) were grown to a stationary phase, and their cell density was adjusted to OD_600_ ≈ 1.5 with trypticase soy broth. Next, WC cultures were preincubated with 2 mM 2,2′-dithiodipyridine for 15 min and centrifuged (5000×*g*; 20 min) to separate the cell-free culture medium, which was designated ‘supernatant fraction’ (S). The resulting cell pellets were washed and resuspended in ice-cold phosphate-buffered saline (PBS) supplemented with a complete EDTA-free protease inhibitor mix (Roche) and 0.2 mM tosyl-ʟ-lysyl-chloromethane hydrochloride (Sigma) and subjected to cell disruption at 30 kPa pressure with a BT40/TS2/AA cell disruptor from Constant Systems. This was followed by 30 min digestion with 0.02 mg ml^−1^ DNase I (Roche), which resulted in the ‘cell extract’ (C) fraction. The remaining cell lysate was ultracentrifuged at 150 000×*g* for 1 h and the soluble fraction (‘periplasmic/cytoplasmic’ fraction; PP) was separated from the pellet, which contained the insoluble membrane fraction. IM proteins were solubilized by incubation with 200 mM magnesium chloride and 10% Triton X-100 at 4°C for 30 min and separated by further centrifugation at 150 000×*g* for 1 h. The remaining insoluble pellet contained OM proteins (‘OM fraction’) and was resuspended by sonication in PBS supplemented with the above protease inhibitors. Protein concentrations were determined with the BCA protein assay kit from Thermo Fisher Scientific, and fractions were stored at −20°C.

### 3.3. Generation of C-terminal domain mutants

Genomic modification of *P. gingivalis* was performed by homologous recombination with suicide plasmids carrying an antibiotic resistance cassette, as described before [41]. Primers used for PCR reactions are listed in table 4. For RgpB and PorU mutagenesis, the two master plasmids pRgpB-t [16] and p22-E-1158iHis [41] were used, respectively. The desired mutations (RgpB: A^719^R, 695-701:GG and G^699^R; PorU:1152sHis) were introduced into the plasmids by standard PCR- and ligase-based methods. For RgpB mutant 712-714:NARGDGNSVA, subcloning of a commercially synthesized 542 bp long fragment of the RgpB gene carrying the desired substitution was performed.

**Table 4.** List of vectors and primers (5’→3’) used for mutant generation.

***pPorU1152sHis***	
P22i6h_Fs	TAGCCTCTAGAATAGCTTCCGC
P22i6h_Ft	CACCATCACCATCACCATTAGCCTCTAGAATAGCTTCCGC
P22d1129_Rs	TTTCTTGGCCATGGAGGC
P22d1129_Rt	ATGGTGATGGTGATGGTGTTTCTTGGCCATGGAGGC
***pRgpB695-701:GG***	
RB695GGFnew2	GGCGTAGCTACTGCTAAAAACCGCATGG
RB695GGRnew2	ACCGATCGTCAGCCCGGCAGCA
***pRgpBG699R***	
RgpBG699R_F	GATCTTCGATATGAACCGCCGTGTAGCTACTG
RgpBG699R_R	CAGTAGCTACACGACGGCGGTTCATATCGAAGATC
***pRgpB 712-714:NARGDGNSVA***	
inRBporZ712F	GCAGGTTACGGCTCCGGCAA
inRBporZ712R	GGCTGTGCCGAATGGATTCC
vRBporZ712F	TCCATTCGGCACAGCCCTT
vRBporZ712R	CGGAGCCGTAACCTGCATT
***pRgpB A719R***	
RgpBA719R_F	CAAAACGGCGTGTATCGCGTTCGCATCGCTACTGAAGGC
RgpBA719R _R	GCCTTCAGTAGCGATGCGAACGCGATACACGCCGTTTTG
***pPPAD-tet***	
pUPPADa_F	GATGATGAATTCCCCAAATACGAAGCAC
pUPPADa_R	ATTACCCGGGAATTAAGAATATCAGTGGAG
pUPPADb_F	CAGCGTCGACGGGTTATTATTCAAAATCTGAGC
pUPPADb_R	TCACTGCAGCTCCGTATAGAGCAGGATC
***pPPAD-LKK***	
749_364_inF	CATGTACTGTGACCGGAGATAATCTTTATCTCACCCTCT
750_364_inR	TTCTCAAATAAGGGGCCTCATTTTTTGATCAATTTCTGC
751_364_vR	TCCGGTCACAGTACATGTATT
752_364_vF	GGCCCCTTATTTGAGAATAC
***LKK-pGEX6-P-1***	
LKK-F	TCAGGGATCCCTCACCCTCTCCCCCAACCCGGC
LKK-R	CTGGCTCGAGTTATTTTTTGATCAATTTCTGCGTG
***pWbpB-t***	
wbpB_puc19R	GAATTCACTGGCCGTCGTTTTAC
wbpBupF	CGACGGCCAGTGAATTCTCGTGTTCGGATTGTATGAAC
wbpBupR	GTTAAGGAGATAATTCGTTGTTGTCCTGTTCCTCATTATATCTG
wbpBtetF	ACAACGAATTATCTCCTTAACG
wbpBtetR	GCCAAGTTCTAATGCTTCTATCACAACGAATTATCTCCTTAACG
wbpBdwF	AGAAGCATTAGAACTTGGCAAAAGGAATTCCTATAATCTTATC
wbpBdwR	CATGCCTGCAGGTCGACATGATCAAATTCATGAGTATCGC
wbpB_puc19F	GTCGACCTGCAGGCATGCA

For PPAD mutagenesis, master-plasmid pPPAD-tet was designed based on the pUC19 background to introduce a tetracycline resistance gene just after the coding sequence of the *ppad* gene. The LKK domain was amplified by PCR from *T. forsythia* genomic DNA. The chimaera of *P. gingivalis* PPAD (UP Q9RQJ2; residues M^1^–G^473^) fused with fragment D^702^–K^791^ of *T. forsythia* mirolase was generated by Gibson cloning. A control strain, ∆WbpB, which is a *P. gingivalis* strain that does not glycosylate T9SS cargoes but secretes them without attached lipopolysaccharide [77], was generated based on the pWbpB-t suicide plasmid, itself obtained by Gibson cloning based on pUC19.

Generated plasmids were introduced by electroporation into either wild-type *P. gingivalis* W83 cells (for PorU, PPAD and ΔWbpB modifications) or the ∆*rgpA* strain of *P. gingivalis* W83, named RgpA-C (for RgpB modification) and subjected to antibiotic selection. Positive clones were verified by DNA sequencing.

### 3.4. Proteolytic activity assays

Extracellular gingipain activity was measured as described [41,71], with the chromogenic substrates AcO-K-*p*-nitroanilide (for Kgp) or PheCO-R-*p*-nitroanilide (for RgpA/B) at 1 mM in assay buffer (200 mM Tris·HCl, 100 mM sodium chloride, 5 mM calcium chloride, 10 mM ʟ-cysteine and pH 7.6) at 37°C in triplicate. The change in absorbance was monitored at *λ* = 405 nm in a SpectroMax M5 microplate reader (Molecular Devices). Enzyme activity was presented as a percentage of the control strain activity.

### 3.5. Peptidyl arginine deiminase activity

PPAD activity was assayed with *N*-acetylarginine as the substrate (at 10 mM) in 100 mM Tris·HCl, 2.5 mM dithiothreitol, pH 7.5, as reported [78]. Results were obtained from three independent biological replicates for whole-culture or cell-free-media samples and presented as per cent of the activity of the *P. gingivalis* wild-type strain. The results of strain ΔWbpB were further presented for completeness.

### 3.6. Western blot analyses

The distinct subcellular fraction samples of §3.2 were resolved by SDS-PAGE and blotted on nitrocellulose membranes. Unspecific binding was ablated with blocking buffer (5% non-fat skim milk in PBS *plus* 0.05% (v/v) Tween-20). The primary antibodies mAb 7G9 (for the detection of PorU), as well as pAb GP-1 and anti-CTD (for the detection of RgpB), were diluted in blocking buffer and incubated overnight at 4°C with the blotted samples. Anti-mouse (for anti-PorU blots) or anti-rabbit (for anti-RgpB and anti-CTD blots) horseradish-peroxidase-conjugated IgGs from goat were used as secondary antibodies at 1:20 000 dilution.

### 3.7. LKK protein production and purification

The coding sequence of LKK, whose boundaries (L^706^–K^791^) were designed before the structure of the first CTD was available, was amplified by PCR from genomic DNA of *T. forsythia* strain ATCC 43037 with forward primer LKK-F and reverse primer LKK-R (see table 4). The amplicon was purified and cloned using *Bam*HI and *Xho*I sites into the pGEX-6P-1 expression vector, which attaches an N-terminal glutathione-S-transferase fusion protein for affinity purification followed by a recognition sequence for PreScission peptidase. The plasmid was verified by sequencing and transformed into *E. coli* strain BL21 (DE3) for protein expression. In order to obtain a heavy-atom derivative of the protein, which includes three methionines in its sequence, for structure solution by the SAD method, bacteria were grown in SelenoMethionine complete medium supplemented with SelenoMethionine solution (both from Molecular Dimensions) at 37°C to OD_600_ = 0.5 and induced with 0.5 mM isopropyl-1-thio-β-ᴅ-galactopyranoside. After 4 h cultivation at 37°C, cells were harvested by centrifugation (6000×*g*, 15 min, 4°C) and resuspended in 15 ml wash buffer (PBS with 0.02% sodium azide, pH 7.3) per pellet from 1 l cell culture, and subsequently lysed by sonication (30 × 0.5 s pulses at 70% maximal amplitude per pellet from 1 l culture) using a Branson Sonifier Digital 450 cell disrupter (Branson Ultrasonics). Cell lysates were clarified by centrifugation (50 000×*g*, 30 min, 4°C) and applied at 4°C onto a glutathione-Sepharose 4 Fast Flow column (Cytiva) previously equilibrated with wash buffer. After washing out unbound proteins, the protein was released by in-column digestion with PreScission peptidase in wash buffer (0.01 U ml^−1^) for 40 h at 4°C. The final protein contained the extra N-terminal residues G^−4^–P–L–G–S^−1^ from the tag. Protein concentrations were determined through OD_280_ measured in a NanoDrop microvolume spectrophotometer (Thermo Fisher Scientific) using the Beer–Lambert law and through the BCA protein assay kit, and protein purity was monitored by SDS-PAGE.

### 3.8. Crystallization and X-ray diffraction data collection

The selenomethionine derivative of LKK was crystallized by the sitting-drop vapour diffusion method. Reservoir solutions were prepared in 96-deep-well blocks of 2 ml capacity with a Tecan robot. A Phoenix robot (Art Robbins) dispensed drops of 100 nl or 200 nl protein solution (at 19 mg ml^−1^ in 5 mM Tris·HCl, pH 8.0) and 100 nl reservoir solution into 96-well 2-drop Swissci PS MRC plates (Molecular Dimensions). Over 1500 conditions from different screenings were assayed at the in-house Automated Crystallography Platform (https://www.ibmb.csic.es/en/platforms/automated-crystallographic-platform). Plates were stored at 4°C or 20°C in Bruker steady-temperature crystal farms with remote access for crystal-growth monitoring. The morphologically best crystals were obtained from drops with 30% polyethylene glycol 2000, 0.1 M potassium thiocyanide as reservoir solution (figure 3*a*).

Crystals were cryo-protected by rapid passage through drops containing 40% polyethylene glycol 2000, 10% glycerol (v/v) and flash vitrified in liquid nitrogen before transport in a liquid-nitrogen cryogenic dewar to the ESRF synchrotron in Grenoble (France). Diffraction data were collected to 1.6 Å resolution from a crystal cryocooled at −173°C on an ADSC Quantum Q315r CCD detector at beam-line ID23-1 on 28 August 2011. The chosen wavelength corresponded to the selenium absorption edge, which was determined by a previous X-ray absorption near-edge fluorescence scan. The data were indexed, integrated, and merged with programs *Xds* [73] and *Xscale* [79], as well as *Combat*/*Scala* [80] from the *Ccp4* suite of programs [81]. Data were transformed with *Xdsconv* or *Truncate* [82] to *Mtz*- and *Shelx*-format for structure solution and refinement. Table 2 provides a summary of the data processing statistics.

### 3.9. Structure solution and refinement

Initial automatic indexing suggested that the space group was R32, which led to very good processing statistics and an overall R_meas_ of 7.7%. However, with this setting, only 65% of the collected spots were used for indexing and the structure could not be solved by SAD despite the presence of significant anomalous differences in the data. Thus, the data were reprocessed with space group P3_1_21, which employed all the spots collected for indexing and yielded an overall R_meas_ of 10.6%. With these data, the structure was solved by SAD with the program pipeline *Xprep-Shelxd-Shelxe* [83,84], which found all nine selenium sites in the a.u., enabled distinction between the space group and its enantiomorph and yielded a set of phases for automatic tracing with *Arp*/*wArp* [85]. The resulting model, consisting of three protomers per a.u., was completed through manual remodelling with the *Coot* program [86], but the final map was partially blurry and contained several regions with spurious density. Not surprisingly, crystallographic refinement of the model with the *Refine* protocol of the *Phenix* suite [87] stalled at R_factor_/free R_factor_ values of ~35%/40%.

Thus, the data were reprocessed with space group P3_1_. Analysis with *Xtriage* within *Phenix* revealed a strong off-origin peak of 72% height relative to the origin peak at fractional cell coordinates 0.331, 0.663 and 0.678, which was indicative of strong tNCS (figure 3*b*). Moreover, the data were hemihedrally twinned according to twin law (h, –h–k and –l). The values of the twin fraction *α* estimated by *Xtriage* ranged from 0.423 (ML α) to 0.430 (Britton) to 0.478 (H α). To determine the correct value, data from *Xds* processing were prepared for *Ccp4* with *Combat* and *Sortmtz*, scaled with *Scala* and systematically detwinned with *Detwin* (https://www.ccp4.ac.uk/html/detwin.html) testing *α* values ranging from 0.30 to 0.48. The resulting data were transformed to *Cns*-format with *Mtz2various* within *Ccp4* and analysed with the Padilla & Yeates twinning procedure (https://srv.mbi.ucla.edu/Twinning) [88], which determines local intensity differences by the formula L=(I_1_–I_2_)/(I_1_+I_2_), with I_1_ and I_2_ being the reflection intensities to be compared. This approach is insensitive to anisotropic diffraction and pseudo-centring [88]. The values of <|L|> and <L^2^> for the different twin fractions were compared with the theoretical values of a non-twinned dataset, which are 0.500 and 0.333, respectively. The best match was obtained with *α* = 0.423 (<|L|> = 0.493 and <L^2^> = 0.340).

Subsequent attempts to directly solve the structure within space group P3_1_ and untreated data by SAD, as aforementioned for P3_1_21, failed. By contrast, molecular replacement calculations with *Phaser* using a monomer of the model built into the P3_1_21 Fourier map, with the program option Tncs inactivated, unambiguously found six molecules in the a.u. However, automatic crystallographic refinement of this model with *Refine* within *Phenix* or *Shelxl*, with the twinning option on, determined *α* to be ~0.5 in both cases, and yielded poor Fourier maps and R values. In contrast, refinement with programs *Phenix* and *Buster/Tnt* [89] with standard parameters for non-twinned data against the dataset detwinned as aforementioned with *α* = 0.423 yielded acceptable Fourier maps and enabled completion and refinement of the model of LKK to final R_factor_/free R_factor_ values of 27.5%/30.2%. This model encompassed residues L^706^–K^791^ *plus* two N-terminal tag residues (S^−1^+G^−2^) in six copies per a.u. (molecules A–F) *plus* six chloride and two potassium ions, six diethylene glycol molecules and 476 solvent molecules (see table 2). While these R values are generally high for the resolution of the data, they are deemed acceptable given the intrinsic difficulty of handling data with (i) strong tNCS (molecules D–F result from pure translations of A–C); (ii) strong hemihedral twinning; and (iii) six protein monomers in the a.u. Moreover, all other validation parameters of the final model are within usually acceptable limits (table 2).

### 3.10. Miscellaneous

Figures were prepared with the *Chimera* program [90]. Secondary structure predictions were calculated with *Jpred4* [91], and structure-similarity searches were performed with *Dali* [75]. Structure-based sequence alignments were first calculated with *Promals3d* (https://prodata.swmed.edu/promals3d) [92] and subsequently manually adjusted based on structure superposition, which was performed with the *Ssm* routine [93] within *Coot* and secondary structure predictions. Superposition of structures was also performed with *Chimera* and *PDBeFold* [94].

Comparative models for the CTDs of PPAD (K^476^–K^556^; UP A0A1R4AEC9) and CPG70 (Q^746^–G^821^; UP A0A1R4AHF7) were calculated with *AlphaFold* [95] using paired multiple sequence alignments, which enable the extraction of coevolutionary information and enhance the prediction accuracy [96]. The local confidence of the models was assessed by means of the predicted local-distance difference test (pLDDT) [97], which reliably estimates the accuracy of the Cα local-distance difference test [95]. In this respect, pLDDT values >90% account for high accuracy of the overall prediction and values >70% indicate generally correct predictions of the backbone [98]. The predicted CTD models of CPG70 and PPAD evince average pLDDT values of 93% and 92%, respectively, which indicates they are highly accurate.

## Data Availability

All data and reagents developed for the work presented in this paper can be obtained from the authors upon reasonable request. The new experimental crystal structure of the paper has been submitted to the Protein Data Bank and is universally accessible under code 8QB1. Supplementary material is available online [99].

## References

[1] Economou A, Christie PJ, Fernandez RC, Palmer T, Plano GV, Pugsley AP. 2006 Secretion by numbers: protein traffic in prokaryotes. Mol. Microbiol. **62**, 308–319. (10.1111/j.1365-2958.2006.05377.x) 17020575 PMC3873778

[2] Christie PJ. 2019 The rich tapestry of bacterial protein translocation systems. Protein J. **38**, 389–408. (10.1007/s10930-019-09862-3) 31407127 PMC6826261

[3] Trivedi A, Gosai J, Nakane D, Shrivastava A. 2022 Design principles of the rotary type 9 secretion system. Front. Microbiol. **13**, 845563. (10.3389/fmicb.2022.845563) 35620107 PMC9127263

[4] Paillat M, Lunar Silva I, Cascales E, Doan T. 2023 A journey with type IX secretion system effectors: selection, transport, processing and activities. Microbiology (Reading) **169**, 001320. (10.1099/mic.0.001320) 37043368 PMC10202324

[5] Lasica AM *et al*. 2016 Structural and functional probing of PorZ, an essential bacterial surface component of the type-IX secretion system of human oral-microbiomic Porphyromonas gingivalis. Sci. Rep. **6**, 37708. (10.1038/srep37708) 27883039 PMC5121618

[6] McBride MJ, Zhu Y. 2013 Gliding motility and Por secretion system genes are widespread among members of the phylum bacteroidetes. J. Bacteriol. **195**, 270–278. (10.1128/JB.01962-12) 23123910 PMC3553832

[7] Sato K, Naito M, Yukitake H, Hirakawa H, Shoji M, McBride MJ, Rhodes RG, Nakayama K. 2010 A protein secretion system linked to bacteroidete gliding motility and pathogenesis. Proc. Natl Acad. Sci. USA **107**, 276–281. (10.1073/pnas.0912010107) 19966289 PMC2806738

[8] Goulas T *et al*. 2015 Structure and mechanism of a bacterial host-protein citrullinating virulence factor, Porphyromonas gingivalis peptidylarginine deiminase. Sci. Rep. **5**, 11969. (10.1038/srep11969) 26132828 PMC4487231

[9] Lasica AM, Ksiazek M, Madej M, Potempa J. 2017 The Type IX Secretion System (T9SS): highlights and recent insights into its structure and function. Front. Cell. Infect. Microbiol. **7**, 215. (10.3389/fcimb.2017.00215) 28603700 PMC5445135

[10] Gupta RS, Lorenzini E. 2007 Phylogeny and molecular signatures (conserved proteins and indels) that are specific for the bacteroidetes and chlorobi species. BMC Evol. Biol. **7**, 71. (10.1186/1471-2148-7-71) 17488508 PMC1887533

[11] Kulkarni SS, Zhu Y, Brendel CJ, McBride MJ. 2017 Diverse C-terminal sequences involved in Flavobacterium johnsoniae protein secretion. J. Bacteriol. **199**, e00884–00816. (10.1128/JB.00884-16) 28396348 PMC5446621

[12] Rinke C *et al*. 2013 Insights into the phylogeny and coding potential of microbial dark matter. Nature **499**, 431–437. (10.1038/nature12352) 23851394

[13] Bochtler M, Mizgalska D, Veillard F, Nowak ML, Houston J, Veith P, Reynolds EC, Potempa J. 2018 The Bacteroidetes Q-rule: pyroglutamate in signal peptidase I substrates. Front. Microbiol. **9**, 230. (10.3389/fmicb.2018.00230) 29545777 PMC5837995

[14] Raut MP, Couto N, Karunakaran E, Biggs CA, Wright PC. 2019 Deciphering the unique cellulose degradation mechanism of the ruminal bacterium Fibrobacter succinogenes S85. Sci. Rep. **9**, 16542. (10.1038/s41598-019-52675-8) 31719545 PMC6851124

[15] Williams TJ, Allen MA, Berengut JF, Cavicchioli R. 2021 Shedding light on microbial ‘dark matter’: insights into novel Cloacimonadota and Omnitrophota from an Antarctic lake. Front. Microbiol. **12**, 741077. (10.3389/fmicb.2021.741077) 34707591 PMC8542988

[16] Nguyen KA, Travis J, Potempa J. 2007 Does the importance of the C-terminal residues in the maturation of RgpB from Porphyromonas gingivalis reveal a novel mechanism for protein export in a subgroup of Gram-negative bacteria? J. Bacteriol. **189**, 833–843. (10.1128/JB.01530-06) 17142394 PMC1797278

[17] Veith PD, Nor Muhammad NA, Dashper SG, Likić VA, Gorasia DG, Chen D, Byrne SJ, Catmull DV, Reynolds EC. 2013 Protein substrates of a novel secretion system are numerous in the Bacteroidetes phylum and have in common a cleavable C-terminal secretion signal, extensive post-translational modification, and cell-surface attachment. J. Proteome Res. **12**, 4449–4461. (10.1021/pr400487b) 24007199

[18] Narita Y, Sato K, Yukitake H, Shoji M, Nakane D, Nagano K, Yoshimura F, Naito M, Nakayama K. 2014 Lack of a surface layer in Tannerella forsythia mutants deficient in the type IX secretion system. Microbiology **160**, 2295–2303. (10.1099/mic.0.080192-0) 25023245 PMC4175972

[19] Tomek MB, Neumann L, Nimeth I, Koerdt A, Andesner P, Messner P, Mach L, Potempa JS, Schäffer C. 2014 The S-layer proteins of Tannerella forsythia are secreted via a type IX secretion system that is decoupled from protein O-glycosylation. Mol. Oral Microbiol. **29**, 307–320. (10.1111/omi.12062) 24943676 PMC4232474

[20] Zhu Y, McBride MJ. 2014 Deletion of the Cytophaga hutchinsonii type IX secretion system gene sprP results in defects in gliding motility and cellulose utilization. Appl. Microbiol. Biotechnol. **98**, 763–775. (10.1007/s00253-013-5355-2) 24257839

[21] Kita D, Shibata S, Kikuchi Y, Kokubu E, Nakayama K, Saito A, Ishihara K. 2016 Involvement of the type IX secretion system in Capnocytophaga ochracea gliding motility and biofilm formation. Appl. Environ. Microbiol. **82**, 1756–1766. (10.1128/AEM.03452-15) 26729712 PMC4784043

[22] Li N *et al*. 2017 The type IX secretion system is required for virulence of the fish pathogen Flavobacterium columnare. Appl. Environ. Microbiol. **83**, e01769–e01717. (10.1128/AEM.01769-17) 28939608 PMC5691404

[23] Chen S, Blom J, Loch TP, Faisal M, Walker ED. 2017 The emerging fish pathogen Flavobacterium spartansii isolated from chinook salmon: comparative genome analysis and molecular manipulation. Front. Microbiol. **8**, 2339. (10.3389/fmicb.2017.02339) 29250046 PMC5714932

[24] Kondo Y, Sato K, Nagano K, Nishiguchi M, Hoshino T, Fujiwara T, Nakayama K. 2018 Involvement of PorK, a component of the type IX secretion system, in Prevotella melaninogenica pathogenicity. Microbiol. Immunol. **62**, 554–566. (10.1111/1348-0421.12638) 30028034

[25] Taillefer M, Arntzen MØ, Henrissat B, Pope PB, Larsbrink J. 2018 Proteomic dissection of the cellulolytic machineries used by soil-dwelling Bacteroidetes. mSystems **3**, 00240–00218. (10.1128/mSystems.00240-18) PMC6247017 30505945

[26] Naas AE et al. 2018 ‘Candidatus Paraporphyromonas polyenzymogenes’ encodes multi-modular cellulases linked to the type IX secretion system. Microbiome **6**, 44. (10.1186/s40168-018-0421-8) 29490697 PMC5831590

[27] Sato K *et al*. 2018 Immunoglobulin-like domains of the cargo proteins are essential for protein stability during secretion by the type IX secretion system. Mol. Microbiol. **110**, 64–81. (10.1111/mmi.14083) 30030863

[28] McBride MJ. 2019 Bacteroidetes gliding motility and the type IX secretion system. Microbiol. Spectr. **7**, PSIB-0002-2018. (10.1128/microbiolspec.PSIB-0002-2018) PMC11588200 30767845

[29] Ramos KRM, Valdehuesa KNG, Bañares AB, Nisola GM, Lee WK, Chung WJ. 2020 Overexpression and characterization of a novel GH16 β-agarase (Aga1) from cellulophaga omnivescoria W5C. Biotechnol. Lett. **42**, 2231–2238. (10.1007/s10529-020-02933-x) 32519168

[30] Barbier P, Rochat T, Mohammed HH, Wiens GD, Bernardet JF, Halpern D, Duchaud E, McBride MJ. 2020 The type IX secretion system is required for virulence of the fish pathogen Flavobacterium psychrophilum. Appl. Environ. Microbiol. **86**, e00799–e00720. (10.1128/AEM.00799-20) 32532872 PMC7414955

[31] Chen Z, Niu P, Ren X, Han W, Shen R, Zhu M, Yu Y, Ding C, Yu S. 2022 Riemerella anatipestifer T9SS effector SspA functions in bacterial virulence and defending natural host immunity. Appl. Environ. Microbiol. **88**, e0240921. (10.1128/aem.02409-21) 35575548 PMC9195944

[32] Naito M, Shoji M, Sato K, Nakayama K. 2022 Insertional inactivation and gene complementation of Prevotella intermedia type IX secretion system reveals its indispensable roles in black pigmentation, hemagglutination, protease activity of interpain A, and biofilm formation. J. Bacteriol. **204**, e0020322. (10.1128/jb.00203-22) 35862729 PMC9380532

[33] Astafyeva Y, Gurschke M, Streit WR, Krohn I. 2022 Interplay between the microalgae Micrasterias radians and its symbiont Dyadobacter sp. HH091. Front. Microbiol. **13**, 1006609. (10.3389/fmicb.2022.1006609) 36312980 PMC9606717

[34] Escribano MP, Balado M, Toranzo AE, Lemos ML, Magariños B. 2023 The secretome of the fish pathogen Tenacibaculum maritimum includes soluble virulence-related proteins and outer membrane vesicles. Front. Cell. Infect. Microbiol. **13**, 1197290. (10.3389/fcimb.2023.1197290) 37360528 PMC10288586

[35] Kondo Y, Ohara K, Fujii R, Nakai Y, Sato C, Naito M, Tsukuba T, Kadowaki T, Sato K. 2023 Transposon mutagenesis and genome sequencing identify two novel, tandem genes involved in the colony spreading of Flavobacterium collinsii, isolated from an ayu fish, Plecoglossus altivelis. Front. Cell. Infect. Microbiol. **13**, 1095919. (10.3389/fcimb.2023.1095919) 36844397 PMC9950754

[36] Socransky SS, Haffajee AD, Cugini MA, Smith C, Kent Jr RL. 1998 Microbial complexes in subgingival plaque. J. Clin. Periodontol. **25**, 134–144. 9495612 10.1111/j.1600-051x.1998.tb02419.x

[37] Hajishengallis G *et al*. 2011 Low-abundance biofilm species orchestrates inflammatory periodontal disease through the commensal microbiota and complement. Cell Host Microbe **10**, 497–506. (10.1016/j.chom.2011.10.006) 22036469 PMC3221781

[38] Kassebaum NJ, Bernabé E, Dahiya M, Bhandari B, Murray CJ, Marcenes W. 2014 Global burden of severe periodontitis in 1990-2010: a systematic review and meta-regression. J. Dent. Res. **93**, 1045–1053. (10.1177/0022034514552491) 25261053 PMC4293771

[39] Tonetti MS, Jepsen S, Jin L, Otomo-Corgel J. 2017 Impact of the global burden of periodontal diseases on health, nutrition and wellbeing of mankind: a call for global action. J. Clin. Periodontol. **44**, 456–462. (10.1111/jcpe.12732) 28419559

[40] Butt K, Butt R, Sharma P. 2019 The burden of periodontal disease. Dent. Update **46**, 907–913. (10.12968/denu.2019.46.10.907)

[41] Mizgalska D *et al*. 2021 Intermolecular latency regulates the essential C-terminal signal peptidase and sortase of the Porphyromonas gingivalis type-IX secretion system. Proc. Natl Acad. Sci. USA **118**, e2103573118. (10.1073/pnas.2103573118) 34593635 PMC8501833

[42] Hočevar K, Potempa J, Turk B. 2018 Host cell-surface proteins as substrates of gingipains, the main proteases of Porphyromonas gingivalis. Biol. Chem. **399**, 1353–1361. (10.1515/hsz-2018-0215) 29927743

[43] Bereta G, Goulas T, Madej M, Bielecka E, Solà M, Potempa J, Gomis-Rüth FX. 2019 Structure, function, and inhibition of a genomic/clinical variant of Porphyromonas gingivalis peptidylarginine deiminase. Protein Sci. **28**, 478–486. (10.1002/pro.3571) 30638292 PMC6371208

[44] Chen YY, Cross KJ, Paolini RA, Fielding JE, Slakeski N, Reynolds EC. 2002 CPG70 is a novel basic metallocarboxypeptidase with C-terminal polycystic kidney disease domains from Porphyromonas gingivalis. J. Biol. Chem. **277**, 23433–23440. (10.1074/jbc.M200811200) 11976326

[45] Sato K *et al*. 2005 Identification of a new membrane-associated protein that influences transport/maturation of gingipains and adhesins of Porphyromonas gingivalis. J. Biol. Chem. **280**, 8668–8677. (10.1074/jbc.M413544200) 15634642

[46] Seers CA, Slakeski N, Veith PD, Nikolof T, Chen YY, Dashper SG, Reynolds EC. 2006 The RgpB C-terminal domain has a role in attachment of RgpB to the outer membrane and belongs to a novel C-terminal-domain family found in Porphyromonas gingivalis. J. Bacteriol. **188**, 6376–6386. (10.1128/JB.00731-06) 16923905 PMC1595369

[47] Kharade SS, McBride MJ. 2014 Flavobacterium johnsoniae chitinase ChiA is required for chitin utilization and is secreted by the type IX secretion system. J. Bacteriol. **196**, 961–970. (10.1128/JB.01170-13) 24363341 PMC3957688

[48] Kharade SS, McBride MJ. 2015 Flavobacterium johnsoniae PorV is required for secretion of a subset of proteins targeted to the type IX secretion system. J. Bacteriol. **197**, 147–158. (10.1128/JB.02085-14) 25331433 PMC4288674

[49] de Diego I *et al*. 2016 The outer-membrane export signal of Porphyromonas gingivalis type IX secretion system (T9SS) is a conserved C-terminal β-sandwich domain. Sci. Rep. **6**, 23123. (10.1038/srep23123) 27005013 PMC4804311

[50] Veith PD, Glew MD, Gorasia DG, Reynolds EC. 2017 Type IX secretion: the generation of bacterial cell surface coatings involved in virulence, gliding motility and the degradation of complex biopolymers. Mol. Microbiol. **106**, 35–53. (10.1111/mmi.13752) 28714554

[51] Green ER, Mecsas J. 2016 Bacterial secretion systems: an overview. In Virulence mechanisms of bacterial pathogens (eds IT Kudva NA Cornick, PJ Plummer, Q Zhang, TL Nicholson, JP Bannantine, BH Bellaire), pp. 213–219, 5th edn. Washington, DC: ASM Press.

[52] Shoji M, Sato K, Yukitake H, Kondo Y, Narita Y, Kadowaki T, Naito M, Nakayama K. 2011 Por secretion system-dependent secretion and glycosylation of Porphyromonas gingivalis hemin-binding protein 35. PLoS ONE **6**, e21372. (10.1371/journal.pone.0021372) 21731719 PMC3120885

[53] Glew MD *et al*. 2012 PG0026 is the C-terminal signal peptidase of a novel secretion system of Porphyromonas gingivalis. J. Biol. Chem. **287**, 24605–24617. (10.1074/jbc.M112.369223) 22593568 PMC3397888

[54] Glew MD, Veith PD, Chen D, Gorasia DG, Peng B, Reynolds EC. 2017 PorV is an outer membrane shuttle protein for the type IX secretion system. Sci. Rep. **7**, 8790. (10.1038/s41598-017-09412-w) 28821836 PMC5562754

[55] Gorasia DG, Veith PD, Chen D, Seers CA, Mitchell HA, Chen YY, Glew MD, Dashper SG, Reynolds EC. 2015 Porphyromonas gingivalis type IX secretion substrates are cleaved and modified by a sortase-like mechanism. PLoS Pathog. **11**, e1005152. (10.1371/journal.ppat.1005152) 26340749 PMC4560394

[56] Veillard F *et al*. 2019 Proteolytic processing and activation of gingipain zymogens secreted by T9SS of Porphyromonas gingivalis. Biochimie **166**, 161–172. (10.1016/j.biochi.2019.06.010) 31212040 PMC6815250

[57] Curtis MA, Thickett A, Slaney JM, Rangarajan M, Aduse-Opoku J, Shepherd P, Paramonov N, Hounsell EF. 1999 Variable carbohydrate modifications to the catalytic chains of the RgpA and RgpB proteases of Porphyromonas gingivalis W50. Infect. Immun. **67**, 3816–3823. (10.1128/IAI.67.8.3816-3823.1999) 10417143 PMC96659

[58] Shoji M, Sato K, Yukitake H, Kamaguchi A, Sasaki Y, Naito M, Nakayama K. 2018 Identification of genes encoding glycosyltransferases involved in lipopolysaccharide synthesis in Porphyromonas gingivalis. Mol. Oral Microbiol. **33**, 68–80. (10.1111/omi.12200) 28972686

[59] Veith PD, Shoji M, O’Hair RAJ, Leeming MG, Nie S, Glew MD, Reid GE, Nakayama K, Reynolds EC. 2020 Type IX secretion system cargo proteins are glycosylated at the C terminus with a novel linking sugar of the Wbp/Vim pathway. mBio **11**, e01497–e01420. (10.1128/mBio.01497-20) 32873758 PMC7468200

[60] Madej M *et al*. 2021 PorZ, an essential component of the type IX secretion system of Porphyromonas gingivalis, delivers anionic lipopolysaccharide to the PorU sortase for transpeptidase processing of T9SS cargo proteins. mBio **12**, e02262–e02220. (10.1128/mBio.02262-20) 33622730 PMC8545088

[61] Yukitake H, Shoji M, Sato K, Handa Y, Naito M, Imada K, Nakayama K. 2020 PorA, a conserved C-terminal domain-containing protein, impacts the PorXY-SigP signaling of the type IX secretion system. Sci. Rep. **10**, 21109. (10.1038/s41598-020-77987-y) 33273542 PMC7712824

[62] Kulkarni SS, Johnston JJ, Zhu Y, Hying ZT, McBride MJ. 2019 The carboxy-terminal region of Flavobacterium johnsoniae SprB facilitates its secretion by the type IX secretion system and propulsion by the gliding motility machinery. J. Bacteriol. **201**, e00218–e00219, (10.1128/JB.00218-19) 31262839 PMC6755757

[63] Xie S, Tan Y, Song W, Zhang W, Qi Q, Lu X. 2022 N-Glycosylation of a cargo protein C-terminal domain recognized by the type IX secretion system in Cytophaga hutchinsonii affects protein secretion and localization. Appl. Environ. Microbiol. **88**, e0160621, (10.1128/AEM.01606-21) 34644163 PMC8752156

[64] Dorgan B, Liu Y, Wang S, Aduse-Opoku J, Whittaker SB, Roberts MAJ, Lorenz CD, Curtis MA, Garnett JA. 2022 Structural model of a Porphyromonas gingivalis type IX secretion system shuttle complex. J. Mol. Biol **434**, 167871. (10.1016/j.jmb.2022.167871) 36404438

[65] Książek M *et al*. 2023 A unique network of attack, defence and competence on the outer membrane of the periodontitis pathogen Tannerella forsythia. Chem. Sci. **14**, 869–888. (10.1039/d2sc04166a) 36755705 PMC9890683

[66] Bork P, Holm L, Sander C. 1994 The immunoglobulin fold. Structural classification, sequence patterns and common core. J. Mol. Biol. **242**, 309–320. (10.1006/jmbi.1994.1582) 7932691

[67] Halaby DM, Poupon A, Mornon J. 1999 The immunoglobulin fold family: sequence analysis and 3D structure comparisons. Protein Eng. **12**, 563–571. (10.1093/protein/12.7.563) 10436082

[68] Berisio R, Ciccarelli L, Squeglia F, De Simone A, Vitagliano L. 2012 Structural and dynamic properties of incomplete immunoglobulin-like fold domains. Protein Pept. Lett. **19**, 1045–1053. (10.2174/092986612802762732) 22533620

[69] Slakeski N, Seers CA, Ng K, Moore C, Cleal SM, Veith PD, Lo AW, Reynolds EC. 2011 C-terminal domain residues important for secretion and attachment of RgpB in Porphyromonas gingivalis. J. Bacteriol. **193**, 132–142. (10.1128/JB.00773-10) 20971915 PMC3019955

[70] Zhou XY, Gao JL, Hunter N, Potempa J, Nguyen KA. 2013 Sequence-independent processing site of the C-terminal domain (CTD) influences maturation of the RgpB protease from Porphyromonas gingivalis. Mol. Microbiol. **89**, 903–917. (10.1111/mmi.12319) 23869473 PMC3773331

[71] Veillard F, Potempa B, Guo Y, Ksiazek M, Sztukowska MN, Houston JA, Koneru L, Nguyen KA, Potempa J. 2015 Purification and characterisation of recombinant His-tagged RgpB gingipain from Porphymonas gingivalis. Biol. Chem. **396**, 377–384. (10.1515/hsz-2014-0304) 25720118 PMC4682895

[72] Einspahr HM, Weiss MS. 2012 Quality indicators in macromolecular crystallography: definitions and applications. In International tables for crystallography. Volume F: crystallography of biological macromolecules (eds E Arnold, DM Himmel, MG Rossmann), pp. 64–74, 2nd edn. Hoboken, NJ: John Wiley & Sons, Inc.

[73] Kabsch W. 2010 XDS. Acta Crystallogr. D **66**, 125–132. (10.1107/S0907444909047337) 20124692 PMC2815665

[74] Ksiazek M, Mizgalska D, Eick S, Thøgersen IB, Enghild JJ, Potempa J. 2015 KLIKK proteases of Tannerella forsythia: putative virulence factors with a unique domain structure. Front. Microbiol. **6**, 312. (10.3389/fmicb.2015.00312) 25954253 PMC4404884

[75] Holm L, Laiho A, Törönen P, Salgado M. 2023 DALI shines a light on remote homologs: one hundred discoveries. Protein Sci. **32**, e4519. (10.1002/pro.4519) 36419248 PMC9793968

[76] Bao K, Belibasakis GN, Thurnheer T, Aduse-Opoku J, Curtis MA, Bostanci N. 2014 Role of Porphyromonas gingivalis gingipains in multi-species biofilm formation. BMC Microbiol. **14**, 258. (10.1186/s12866-014-0258-7) 25270662 PMC4189655

[77] Shoji M, Sato K, Yukitake H, Naito M, Nakayama K. 2014 Involvement of the Wbp pathway in the biosynthesis of Porphyromonas gingivalis lipopolysaccharide with anionic polysaccharide. Sci. Rep. **4**, 5056. (10.1038/srep05056) 24852504 PMC4031482

[78] Boyde TR, Rahmatullah M. 1980 Optimization of conditions for the colorimetric determination of citrulline, using diacetyl monoxime. Anal. Biochem. **107**, 424–431. (10.1016/0003-2697(80)90404-2) 7435971

[79] Kabsch W. 2010 Integration, scaling, space-group assignment and post-refinement. Acta Crystallogr. D **66**, 133–144. (10.1107/S0907444909047374) 20124693 PMC2815666

[80] Evans P. 2006 Scaling and assessment of data quality. Acta Crystallogr. D **62**, 72–82. (10.1107/S0907444905036693) 16369096

[81] Agirre J *et al*. 2023 The CCP4 suite: integrative software for macromolecular crystallography. Acta Crystallogr. D **79**, 449–461. (10.1107/S2059798323003595) PMC10233625 37259835

[82] French S, Wilson K. 1978 On the treatment of negative intensity observations. Acta Crystallogr. A **34**, 517–525. (10.1107/S0567739478001114)

[83] Sheldrick GM. 2010 Experimental phasing with SHELXC/D/E: combining chain tracing with density modification. Acta Crystallogr. D **66**, 479–485. (10.1107/S0907444909038360) 20383001 PMC2852312

[84] Sheldrick GM. 2011 The SHELX approach to experimental phasing of macromolecules. Acta Crystallogr. A **67**, C13. (10.1107/S0108767311099727) PMC5947774 29533236

[85] Langer GG, Hazledine S, Wiegels T, Carolan C, Lamzin VS. 2013 Visual automated macromolecular model building. Acta Crystallogr. D **69**, 635–641. (10.1107/S0907444913000565) 23519672 PMC3606041

[86] Casañal A, Lohkamp B, Emsley P. 2020 Current developments in coot for macromolecular model building of electron cryo-microscopy and crystallographic data. Protein Sci. **29**, 1069–1078. (10.1002/pro.3791) 31730249 PMC7096722

[87] Liebschner D *et al*. 2019 Macromolecular structure determination using X-rays, neutrons and electrons: recent developments in Phenix. Acta Crystallogr. D **75**, 861–877. (10.1107/S2059798319011471) PMC6778852 31588918

[88] Padilla JE, Yeates TO. 2003 A statistic for local intensity differences: robustness to anisotropy and pseudo-centering and utility for detecting twinning. Acta Crystallogr. D **59**, 1124–1130. (10.1107/S0907444903007947) 12832754

[89] Smart OS, Womack TO, Flensburg C, Keller P, Paciorek W, Sharff A, Vonrhein C, Bricogne G. 2012 Exploiting structure similarity in refinement: automated NCS and target-structure restraints in BUSTER . Acta Crystallogr. D **68**, 368–380. (10.1107/S0907444911056058) 22505257 PMC3322596

[90] Pettersen EF, Goddard TD, Huang CC, Couch GS, Greenblatt DM, Meng EC, Ferrin TE. 2004 UCSF Chimera: a visualization system for exploratory research and analysis. J. Comput. Chem. **25**, 1605–1612. (10.1002/jcc.20084) 15264254

[91] Drozdetskiy A, Cole C, Procter J, Barton GJ. 2015 JPred4: a protein secondary structure prediction server. Nucl. Acids Res. **43**, W389–W394. (10.1093/nar/gkv332) 25883141 PMC4489285

[92] Pei J, Kim BH, Tang M, Grishin NV. 2007 PROMALS web server for accurate multiple protein sequence alignments. Nucl. Acids Res. **35**, W649–W652. (10.1093/nar/gkm227) 17452345 PMC1933189

[93] Krissinel E, Henrick K. 2004 Secondary-structure matching (SSM), a new tool for fast protein structure alignment in three dimensions. Acta Crystallogr. D **60**, 2256–2268. (10.1107/S0907444904026460) 15572779

[94] Krissinel E, Henrick K. 2003 Protein structure comparison in 3D based on secondary structure matching (Pdbefold) followed by Cα alignment, scored by a new structural similarity function. In 5th Int. Conf. on Molecular Structural Biology, Vienna, Austria, 3–7 September 2003.

[95] Jumper J *et al*. 2021 Highly accurate protein structure prediction with AlphaFold. Nature **596**, 583–589. (10.1038/s41586-021-03819-2) 34265844 PMC8371605

[96] Bryant P, Pozzati G, Elofsson A. 2022 Improved prediction of protein-protein interactions using AlphaFold2. Nat. Commun. **13**, 1265. (10.1038/s41467-022-28865-w) 35273146 PMC8913741

[97] Mariani V, Biasini M, Barbato A, Schwede T. 2013 lDDT: a local superposition-free score for comparing protein structures and models using distance difference tests. Bioinformatics **29**, 2722–2728. (10.1093/bioinformatics/btt473) 23986568 PMC3799472

[98] Tunyasuvunakool K *et al*. 2021 Highly accurate protein structure prediction for the human proteome. Nature **596**, 590–596. (10.1038/s41586-021-03828-1) 34293799 PMC8387240

[99] Mizgalska D, Rodríguez-Banqueri A, Veillard F, Książęk M, Goulas T, Guevara T *et al*. 2024 Supplementary material from: Structural and functional insights into the C-terminal signal domain of the Bacteroidetes type-IX secretion system. Figshare (10.6084/m9.figshare.c.7272136) PMC11285876 38862016

